# Computed Tomographic Features and Prevalence of Orbital Ligament Mineralization in Dogs

**DOI:** 10.3390/ani15243522

**Published:** 2025-12-05

**Authors:** Ying-Ying Lo, Amélie Montenon, Aurélien Jeandel, Anne-Sophie Bedu

**Affiliations:** 1 Diagnostic Imaging Unit, Centre Hospitalier Vétérinaire AniCura Pommery, 226 Boulevard Pommery, 51100 Reims, France; 2 Neurology Unit, Centre Hospitalier Vétérinaire AniCura Pommery, 226 Boulevard Pommery, 51100 Reims, France

**Keywords:** orbital ligament, mineralization, computed tomography, dog, orbit

## Abstract

Mineralization within the orbital ligament (OL) is occasionally observed on canine head computed tomography (CT) examinations, typically without associated clinical signs. This feature has been only briefly mentioned in the veterinary literature. The present retrospective descriptive study evaluated 402 dogs to determine the prevalence and CT characteristics of OL mineralization, including its location, morphology, margins, symmetry, size, and attenuation. Associations with signalment, medical history and concurrent mineralization were also assessed. Orbital ligament mineralization was identified in 157 of 402 dogs (39.1%). The lesion was consistently located dorsally (100%), and was most often symmetrical, triangular, well-defined and heterogenous. The presence of OL mineralization was significantly associated with increasing age and body weight, as well as with concurrent mineralization in other sites, such as lungs and ears. The lesion was significantly less frequent in brachycephalic dogs. No associations were found with facial trauma, orbital disease or other pathological conditions. Orbital ligament mineralization appears to be a common incidental finding in canine head CT studies, most likely representing a benign, age-related, and non-pathological change.

## Simple Summary

During their clinical practice, the authors frequently noticed a small mineralized focus within the orbital ligament (OL) on canine head computed tomography (CT) scans. Only a single reference in the veterinary literature has previously mentioned this finding, without providing details on its prevalence or clinical relevance. This study aimed to assess the prevalence and characteristics of OL mineralization in dogs based on a retrospective evaluation of 402 head CT scans. Orbital ligament mineralization was identified in 39.1% of the cases. It was consistently located dorsally, and was most often symmetrical, triangular in shape, well-defined and heterogenous in appearance. Statistical analysis revealed a significant association between the presence of OL mineralization and increasing age and body weight, as well as with concurrent mineralization in other anatomical sites. No pathological condition was associated with this lesion. These results suggest that OL mineralization appears to be a common incidental finding in canine head CT studies. It most likely represents a benign, age-related, non-pathological process. Recognizing this feature can help avoid misinterpretation as a pathological lesion during CT evaluation of canine patients.

## 1. Introduction

In dogs, the orbit separates the eye from the cranial cavity and provides protection while allowing passage of vascular and nervous structures through multiple foramina and fissures [1]. Orbital soft tissues include the lacrimal and zygomatic salivary glands, extraocular muscles, and the optic nerve, which are all surrounded by the periorbita and variable amounts of orbital fat [1,2].

The bony orbit is formed by the frontal, lacrimal, maxillary, sphenoid, palatine and zygomatic bones. As the orbit is not a fully enclosed space in dogs and cats, the orbital ligament laterally bridges the frontal process of the zygomatic bone and the zygomatic process of the frontal bone [1,2].

Computed tomography provides a detailed multiplanar assessment of the orbit and is widely used to investigate orbital diseases [3,4]. On CT, the orbital ligament appears as a hyperattenuating soft-tissue structure [5]. The authors have frequently noted the mineralization of this ligament on CT, typically bilateral, well defined, and mildly heterogenous. Aside from one report describing this finding as common [5], no additional veterinary literature exists, and its clinical significance remains unclear.

The primary aim of this study was to describe the CT characteristics and prevalence of orbital ligament mineralization across breeds, body weights, sexes and ages. The secondary aim was to assess any association with clinical or physiological parameters. Given its frequent detection on CT, the authors hypothesized that orbital ligament mineralization represents an incidental, non-pathological finding.

## 2. Materials and Methods

### 2.1. Data Recording

This retrospective, descriptive study was conducted at a single private veterinary hospital (Centre Vétérinaire Hospitalier AniCura Pommery, Reims, France). Canine CT studies and medical records were reviewed from January 2024 to December 2024. All dogs included underwent head CT, with some scans also covering additional regions. No exclusions were made based on sex, age, breed, or body weight.

### 2.2. Image Acquisition

Computed tomography scans were performed using a 64-slice multidetector helical CT scanner (Siemens SOMATOM GO ALL; Erlangen, Germany) with variable kVp and mAs parameters adjusted to patient size. When available, post-contrast studies were obtained following the intravenous administration of iohexol (600 mg iodine/kg; Omnipaque 350 mg I/mL; GE Healthcare, Amsterdam, The Netherlands). Slice thickness ranged from 0.8 mm to 2 mm, and the image matrix was 512 × 512. Dogs were placed in sternal recumbency and maintained under general anesthesia with isoflurane after induction with diazepam (0.2 mg/kg IV) and propofol titrated to effect (4 mg/kg IV). The field of view was adjusted for each patient, and images were reconstructed using both soft-tissue and bone algorithm.

### 2.3. Image Evaluation

Computed tomography studies were reviewed by the first author (Y.Y.L., third-year ECVDI resident) under the supervision of an ECVDI diplomate (A.S.B.). Images were displayed in DICOM format using a medical software (Osirix MD v12.5.3). Evaluation was primarily made on transverse plane, using bone window settings, with window width and level adjustable as needed. Multiplanar reconstructions were also used secondarily.

**Figure 1:**
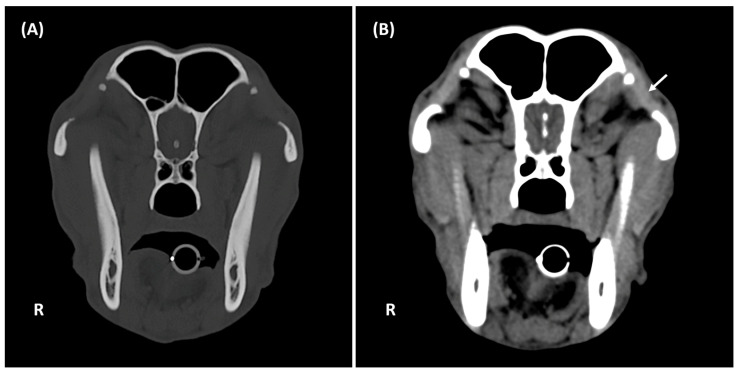
Transverse CT images of the head of an 8-year-old intact female Belgian Malinois (27 kg) showing OL mineralization, displayed with bone window settings (**A**) and soft tissue settings (**B**). The orbital ligament is hyperattenuating (white arrow). The lesion is best delineated using bone window settings (**A**). The contour of the mineralized focus appears less distinct in soft tissue settings (**B**). R: right.

**Figure 2:**
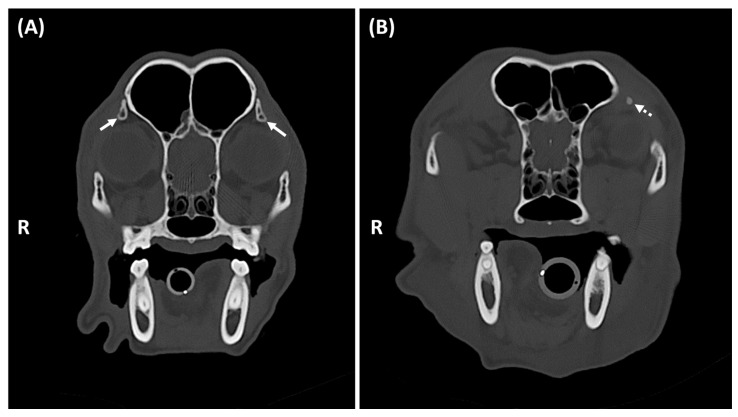
Transverse CT images of the head of two dogs, at the level of the orbit, displayed with bone window settings. In the 8-year-old neutered female Cocker Spaniel (14 kg) shown in (**A**), the OL mineralization is bilateral, symmetrical, triangular, mildly heterogenous and dorsally located (white arrows). In the 8-year-old neutered female Border Collie (36 kg) displayed in (**B**), the OL mineralization is unilateral (dotted arrow). R: right.

**Figure 3:**
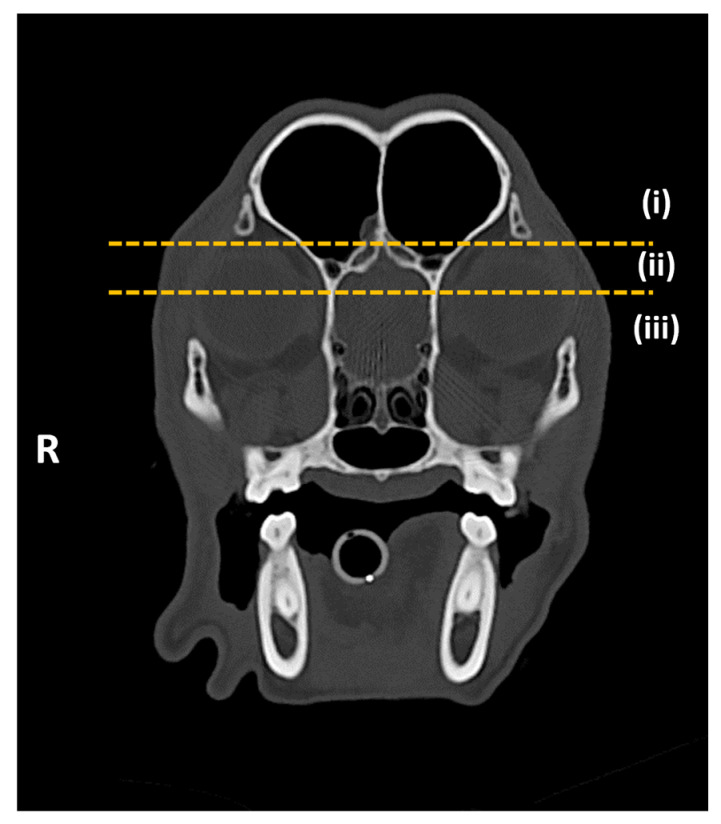
Transverse CT images of the head of the patient shown in Figure 1A, obtained at the level of the orbit, displayed with bone window settings: (i) dorsal position, (ii) central position, and (iii) ventral position, delimited by yellow dotted lines. R: right.

Studies were classified as positive if mineralization was present in at least one orbital ligament and negative if absent (Figure 1). For positive studies, laterality (unilateral, bilateral), symmetry (symmetrical, asymmetrical) (Figure 2), and localization within the orbital ligament (dorsal, central, ventral) were recorded (Figure 3).

**Figure 4:**
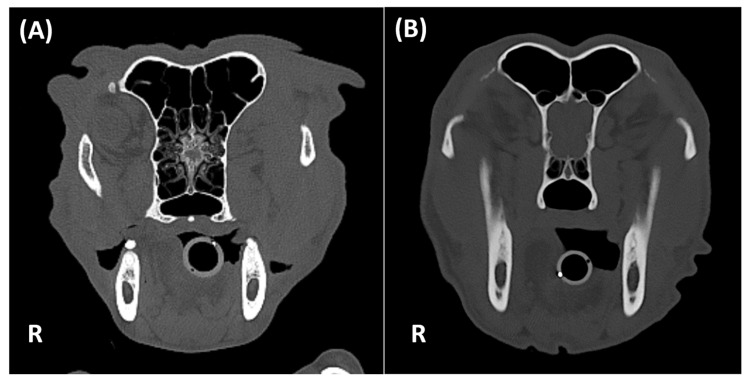

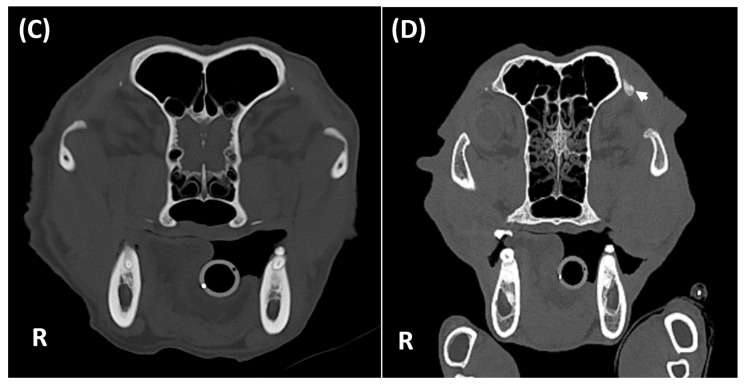
Transverse CT images of the head of different dogs displayed in bone window settings, illustrating various shapes of OL mineralization. (**A**) Nodular-shaped mineralization in a 5-year-old intact male Drathaar (54 kg). (**B**) Linear-shaped mineralization in a 4-year-old intact female Wachtelhund (26 kg). (**C**) Punctate-shaped mineralization in a 11-year-old female neutered Eurasier (28 kg). (**D**) Amorphous mineralization (arrowhead) in a 9-year-old neutered male German Shepherd (49 kg). R: right.

**Figure 5:**
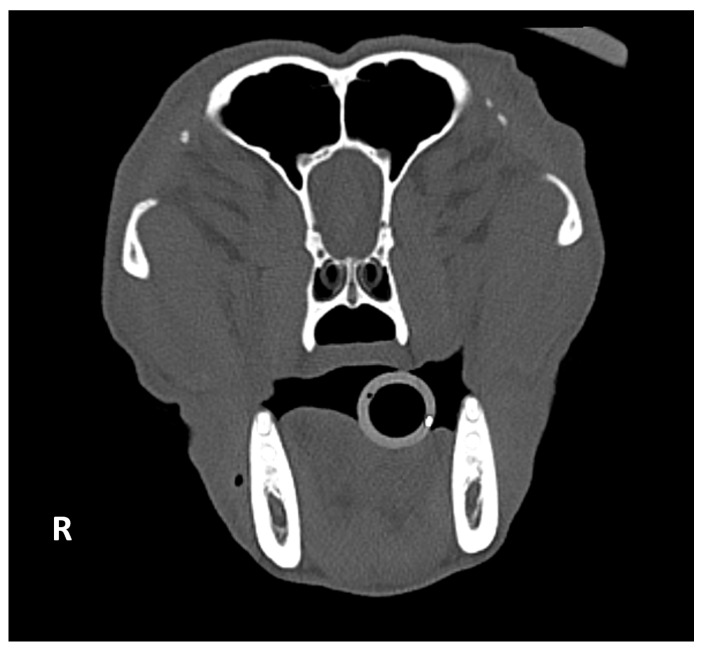
Transverse CT images of the head of a 4-year-old neutered male Siberian Husky (26 kg) showing bilateral OL mineralization, displayed with bone window settings. The mineralization is multifocal on the left side. R: right.

Each lesion was further characterized by its maximum size (mm) using a linear calliper placed along the largest dimension in the transverse section. Maximum attenuation (HU) was obtained using a manually placed circular ROI positioned entirely within the lesion, avoiding partial-volume effects and adjacent bone or soft-tissues. All measurements were performed in standard bone windows (WW: 1500, WL: 300) to ensure consistency. The shape (triangular, nodular, linear, punctate, amorphous) (Figure 4), texture (heterogenous or homogenous), number (focal or multifocal) (Figure 5), and margins (well-defined or ill-defined) were also recorded. Additional mineralization within the head or other regions was also documented.

### 2.4. Clinical Data Analysis

For each dog, the following data were recorded: breed, sex, neuter status, age, and body weight. When available, blood chemistry results, including serum calcium levels were retrieved. Clinical history and physical examination findings were also reviewed. A detailed medical history was examined to identify any evidence of previous facial trauma, orbital or naso-sinusal disease, as well as relevant comorbidities, such as endocrine disorders, chronic corticosteroid administration, or chronic kidney disease. The indication for the CT examination was recorded and categorized as ocular/orbital-related or unrelated.

### 2.5. Statistical Analysis

Analyses were performed using Python (version 3.11), with SciPy library (v1.16.0) for statistical testing and Seaborn (v0.13.2) for data visualization. Categorical variables were analyzed with Chi-square or Fisher’s exact test, depending on the expected frequencies. Continuous and discrete variables were visually assessed for normality using histograms. Normally distributed data were compared using Student’s *t*-test; otherwise, the Mann–Whitney *U* test was applied. Bonferroni correction was used for multiple comparisons. A *p*-value of <0.05 was considered statistically significant.

## 3. Results

### 3.1. Study Population

A total of 402 canine CT studies were included, comprising 175 females and 227 males. Among these, 215 dogs were neutered, 187 intact. Ages ranged from 0.2 to 19 years (median 7 years) and body weights from 2 to 74 kg (median 24 kg).

Orbital ligament mineralization was identified in 157 dogs (39.1%). In this positive study population, 63 were female and 94 males, and 80 were neutered and 77 intact. Ages ranged from 2 to 19 years (median 9 years) and body weights from 6 to 74 kg (median 27 kg).

Positive cases included a variety of breeds, including crossbreed (19/157; 12%), Labrador (15/157; 9%), German Shepherd (15/157; 9%), Australian Shepherd (9/157; 5%), Golden Retriever (9/157; 5%), Cocker Spaniel (7/157; 4%), Belgian Malinois (7/157; 4%), Beagle (5/157; 3%), Brittany Spaniel (5/157; 3%), Jack Russell Terrier (4/157; 2%), Boxer (4/157; 2%), Münsterlander (4/157; 2%), Border Collie (4/157; 2%), French Bulldog (3/157, 1.9%), English Bulldog (3/157; 1.9%), American Staffordshire (3/157; 1.9%), Leonberg (3/157; 1.9%), West Highland White Terrier (3/157; 1.9%), Springer Spaniel (3/157; 1.9%) White Swiss Shepherd (3/157; 1.9%), Griffon (2/157; 1%), Beauceron (2/157; 1%), English Setter (2/157; 1%), German Pointer (2/157; 1%), Pyrenean Mountain Dog (2/157; 1%), Deutsch Drathaar (2/157; 1%), Siberian Husky (2/157; 1%), and 18 other breeds (1/157; 1%). Dogs were further classified as brachycephalic (11/157; 7%), representing 4 different breeds, or non-brachycephalic (146/157; 93%), representing 41 breeds.

The characteristics of the study population parameters are summarized in Table 1.

### 3.2. Imaging Findings

Orbital ligament mineralization was most clearly evaluable on transverse planes using bone window settings (Figure 1). Among 157 positive dogs, 21 (13.4%) were unilateral (14/21; 66,6% right-sided) and 136 (86.6%) bilateral (112/136; 82,4% symmetrical).

A total of 293 lesions were identified. Most mineralization lesions were focal (183/293; 62.5%) versus multifocal (110/293; 37.5%). Morphologically, the majority of lesions was triangular (164; 56.0%), then linear (58; 19.8%), nodular (35; 11.9%), punctate (29; 9.9%), or amorphous (7; 2.4%). Margins were well-defined in 218 cases (74.4%) and ill-defined in 75 (25.6%). Texture was predominantly heterogeneous (191/293; 65.2%), with the remainder homogeneous (102/293; 34.8%). Median lesion size was 2.48 mm (interquartile range [IQR]: 1.52–4.23 mm, range 0.47–9.07 mm) and median attenuation 793 Hounsfield Units (HU) (IQR: 620–988 HU, range 181–1529 HU).

### 3.3. Clinical Data Analysis

Following Bonferroni correction, OL mineralization was significantly associated with age (*p* < 0.0001), weight (*p* = 0.0001), non-brachycephalic phenotype (*p* = 0.0033), non-atrophied frontal sinuses (*p* < 0.0001), lung mineralization (*p* = 0.0015), and number of other mineralization sites (*p* = 0.0008). Marginal association was also observed for ear mineralization (*p* = 0.0409).

Brachycephalic dogs had lower prevalence of OL mineralization (17.2%) than non-brachycephalic (43.2%). Dogs with small/atrophied frontal sinuses were less likely to show OL mineralization (4.3%) than those with normal sinuses (43.7%). Orbital ligament mineralization was more common in dogs with concurrent lung (72.7%) or ear mineralization (47.9%) than those without (36.0% and 32,3%, respectively) (Table 2).

**Figure 6:**
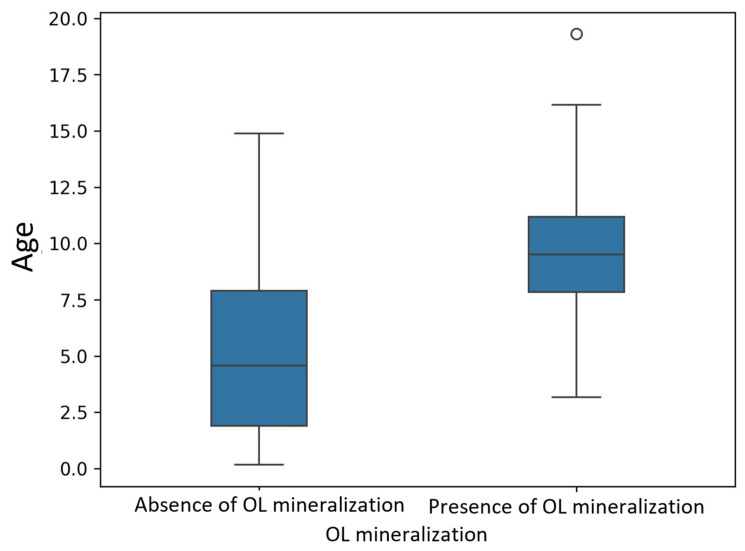
Box plot showing age distribution in dogs, in the presence or absence of OL mineralization.

**Figure 7:**
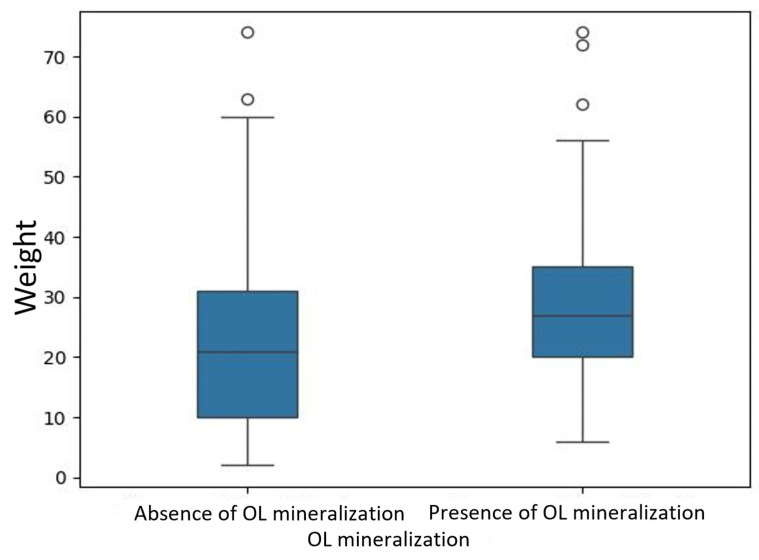
Box plot showing body weights of dogs, in the presence or absence of OL mineralization.

Dogs with OL mineralization were significantly older (median age: 9.0 years; IQR: 7.0–11.0) and heavier (median weight: 27.0 kg; IQR: 20.0–35.0) than dogs without mineralization (median age 4.0 years; IQR: 1.0–7.0; median weight: 21.0 kg; IQR: 10.0–31.0) (Figure 6 and Figure 7). They also had a higher number of other mineralized sites (median: 1.0; IQR: 0.0–1.0) compared to the non-affected group (median: 0.0; IQR: 0.0–1.0).

No significant associations were found with biochemical calcium levels, orbital sinus disease history, head trauma, endocrine disorders, prior steroid administration, chronic kidney disease score, ocular abnormalities, or laryngeal or other non-specific mineralization (*p* > 0.05 for all).

## 4. Discussion

This study reports the frequency and the morphological CT features of OL mineralization in a cohort of 402 dogs. Orbital ligament mineralization was observed in 157 of 402 dogs, corresponding to a prevalence of 39.1% in this population. This rate is higher than previously reported prevalences for other focal soft-tissue mineralization identified in dogs, such as diaphragmatic [6] or supraspinatus entheses mineralization [7].

This mineralization exhibited a relatively uniform morphology, supporting the hypothesis that it represents a normal anatomical or physiologic variant commonly observed in canine head CT studies.

The presence of OL mineralization was significantly associated with increasing age and body weight, suggesting an age-related or degenerative process. Previous reports have described other incidental mineralization in older or heavier dogs [6,8,9], supporting the interpretation of OL mineralization as a common incidental and potentially age-related finding.

Orbital ligament mineralization was further correlated with a higher number of other mineralized sites, particularly in the lungs and, to a lesser extent, in the ears. Fortuitous lung mineralization, often referred to as pulmonary osseous metaplasia is considered a common age-related change [10]. This may account in this study for the frequent co-occurrence of lung and OL mineralization observed in older dogs.

Although a degenerative process has also been hypothesized for ear mineralization [11], it has more commonly been associated with otitis externa and media [12,13]. The correlation between OL and ear mineralization may support a degenerative hypothesis; however, this association could also be influenced by sampling bias, as otic disease is a common indication for performing head CT.

Several forms of benign soft-tissue mineralization have been reported in veterinary medicine [14]. Calcinosis circumscripta is an uncommon syndrome of ectopic, idiopathic, dystrophic, metastatic, or iatrogenic mineralization characterized by calcium salt deposition in soft tissues [15]. It is most frequently observed in the appendicular skeleton but has also been described in facial soft tissues [15]. In this cohort, one dog with OL mineralization was also diagnosed with calcinosis circumscripta in the axillary region, without any additional pathological or biochemical abnormalities. Calcinosis cutis is typically associated with hyperadrenocorticism, or less commonly, with primary or secondary hyperparathyroidism. Metastatic mineralization occurs in normal tissues secondary to systemic disturbances of calcium or phosphate metabolism, such as in renal disease [14]. In this study, the medical history of each patient was reviewed to investigate possible association between endocrine disorders, calcium abnormalities, corticosteroid use, or renal disease and OL mineralization. No significant association was identified, supporting the hypothesis and interpretation of OL mineralization as a benign, incidental, age- and body weight-related, idiopathic, or degenerative finding.

In human medicine, the incidental mineralization of ligaments and soft tissues is well documented [16,17,18]. For example, mineralization of the stylohyoid ligament [16] has been hypothesized to represent an age-related change, either resulting from calcium salt deposition within fibrous tissue or from the direct ossification of residual cartilaginous cells in adults. In the absence of histopathologic evaluation in the present study, such a correlation could not be confirmed.

Mineralization of the trochlear apparatus [17,18] has also been described in humans, particularly following trauma. Similar trauma-associated soft-tissue mineralization and osseous proliferation have been reported in a dog [19] and an African Grey Parrot [20]. A traumatic origin appears unlikely in the case of OL mineralization, given its high prevalence and the absence of any significant association with a history of head trauma.

Hanot et al. [21] previously reported traumatic avulsion of the orbital ligament in three young dogs, with mineral-attenuating foci visible within the orbital ligament on CT. All cases were imaged in the acute phase and no follow-up CT was available, leaving it uncertain whether these lesions could later evolve into degenerative mineralization. In the present study, no correlation was identified between OL mineralization and a history of facial trauma. Interestingly, Hanot et al. [21] hypothesized that immature dogs might have a weaker dorsal attachment of the orbital ligament, as all avulsion sites were located dorsally. In our cohort, all OL mineralization were also dorsally located, potentially supporting this hypothesis. A weaker dorsal attachment could increase ligament mobility and promote focal mineral deposition, as previously suggested in experimental studies [22]. Similar mechanisms related to mechanical strain have been proposed to explain the formation of mineralized bodies in the diaphragm of camels [23].

Orbital ligament mineralization was significantly less frequent in brachycephalic dogs and in those with small or atrophied frontal sinuses. This finding contrasts with the higher prevalence of other incidental mineralization [6] or skeletal anomalies [24] reported in brachycephalic breeds. The reduced frontal sinus size [25], distinct orbital conformation [26,27], and close anatomical relationship between the orbital ligament and the frontal sinuses may contribute to the lower prevalence of OL mineralization in brachycephalic dogs, although the exact pathophysiology remains unknown. The main limitation of this observation is the low number of brachycephalic dogs presenting with OL mineralization (11/157). Despite a reasonable overall representation of brachycephalic breeds, only a small subset showed OL mineralization, limiting the statistical strength of subgroup comparisons. Therefore, the lower prevalence observed in brachycephalic dogs should be interpreted with caution, as the limited sample size may not accurately reflect the true distribution in this population.

To our knowledge, OL mineralization has not been reported in other species. In cats, the processes of the frontal and zygomatic bones extend more toward one another, resulting in a shortened orbital ligament [1]. This anatomical difference may explain the absence of OL mineralization in this species.

Finally, the main limitations of this study include its retrospective design and thus the absence of histopathological analysis, which prevented a definitive characterization of the mineralized tissue, although calcium salt deposition is suspected. Additionally, this study relied on a single primary observer, which also represents a limitation, particularly due to the lack of inter-observer reproducibility assessments. Furthermore, the lack of follow-up CT examinations limited assessment of its progression over time.

## 5. Conclusions

In conclusion, orbital ligament mineralization represents a frequent finding in canine head CT studies, with a prevalence of 39.1% in this cohort. The lesion is consistently dorsally located within the OL, often bilateral and symmetrical, and significantly associated with increasing age, body weight, and the presence of other mineralized sites. No pathological correlation was identified. These findings support the interpretation of OL mineralization as a benign, age-related, and incidental change, without clinical significance.

## Figures and Tables

**Table 1:** Summary of the study population (patients deemed positive for OL mineralization).

Study Population = 157 Patients			
**Sex**	**Number**	**Breeds**	**Number**
Male	94	Crossed	19
Female	63	Labrador	15
		German Shepherd	12
**Status**	**Number**	Golden Retriever	9
Neutered	80	Australian Shepherd	9
Intact	77	Belgian Malinois	7
		Cocker	7
**Age**	**Years**	Brittany Spaniel	5
Minimum	2	Beagle	5
Maximum	19	Jack Russel	4
Median	9	Border Collie	4
		Boxer	4
**Body weights**	**Kg**	Munsterlander	4
Minimum	6	French Bulldog	3
Maximum	74	American Staffordshire Terrier	3
Median	27	Leonberg	3
		English Bulldog	3
		WHWT	3
		Springer Spaniel	3
		White Swiss Shepherd	3
		Beauceron	2
		Draathar	2
		Griffon	2
		German Pointer	2
		Husky	2
		English Setter	2
		Pyrenean Mountain Dog	2
		Chihuahua	1
		Bernese Mountain Dog	1
		Teckel	1
		Chow Chow	1
		Wachtelhund	1
		Coton de Tulear	1
		Bichon	1
		Samoyede	1
		Eurasier	1
		Galgo	1
		Whippet	1
		Saint-Bernard	1
		Belgian Tervueren	1
		Shetland	1
		Basset Hound	1
		Hovawart	1
		Anglos	1
		Basset	1

**Table 2:** Occurrence of OL mineralization in different tested factor groups.

Factor	Group	OL Mineralization (%)
**Skull conformation**	Non-brachycephalic	43.2
	Brachycephalic	17.2
**Frontal sinuses**	Normal	43.7
	Small/Atrophied	4.3
**Lung mineralization**	Present	72.7
	Absent	36
**Ear mineralization**	Present	47.9
	Absent	32.3

## Data Availability

Anonymized dataset is available on request from the authors.

## Abbreviations

The following abbreviations are used in this manuscript:

CT	Computed tomography
OL	Orbital ligament
